# Surgical vs. conservative treatment for hip osteoporotic fracture in maintenance hemodialysis patients: a retrospective analysis

**DOI:** 10.3389/fsurg.2024.1471101

**Published:** 2024-12-06

**Authors:** Man-Yu Zhang, Wei Song, Jing-Bo Wang, Rui-Qian Lv, Fu-Hao Zhao, Ding-Wei Yang

**Affiliations:** ^1^ Department of Nephrology, Tianjin Hospital of Tianjin University, Tianjin, China; ^2^ Department of Hip Traumatology, Tianjin Hospital of Tianjin University, Tianjin, China

**Keywords:** maintenance hemodialysis, osteoporosis, hip fracture, surgical treatment, hip arthroplasty

## Abstract

**Background:**

HSip Osteoporotic fractures are common complications with high mortality in patients undergoing maintenance hemodialysis (MHD). It remains unclear whether surgical or conservative should be adopted for hip fractures in MHD patients.

**Methods:**

A retrospective analysis was conducted in Tianjin Hospital of Tianjin University from August 2019 to August 2023. A total of 43 MHD patients with hip fracture were included, with 30 cases in the surgical group and 13 cases in the conservative group. The differences in cumulative survival rates, time to first ambulation, Harris score, Barthel index, and incidence of complications were compared.

**Results:**

The surgical group had remarkable lower mortality rates as compared with the conservative group at 1, 2, 3, 6, 12, 24 months (13.33 VS. 38.46%, 26.67 VS. 53.85%, 26.67 VS. 53.85%, 26.67 VS. 61.54%, 26.67 VS. 61.54%, and 26.67 VS. 69.23%). In the surgical treatment group, the first ambulation time was reduced to 28 (26) days, which was superior to the conservative group (134.17 ± 43.18 days, *P* < 0.001). The Harris score at 1 month (61.50 ± 4.10) and the Barthel index at 3 months (95, 11.25) were also significantly higher (*P* < 0.001). Furthermore, the surgical group had a significantly lower overall incidence of complications (60.00 vs. 92.31%, *P* = 0.034). The risk of death and complications of surgical treatment was only 23.0 and 32.4% of conservative treatment in MHD patients with hip fracture.

**Conclusion:**

Surgical treatment is effective and safe and should be the first choice for hip fracture in MHD patients.

## 1 Introduction

Osteoporosis is a systemic bone disease characterized by decreased overall bone mass, microstructural damage to bone tissue, increased bone fragility, and a tendency to fracture easily. Based on thecrucial role of kidney in maintaining bone mineral homeostasis and bone remodeling, chronic kidney disease (CKD) further increases the incidence and advances the onset age of osteoporosis. As renal function continues to decline, the risk of fractures can further escalate (1). As the main renal replacement therapy for end-stage renal disease, there are already more than 3.8 million maintenance hemodialysis (MHD) patients globally (2). In MHD patients, osteoporosis and low bone mass are very common. The prevalence rates can reach 9.5%-23% and 16.7%-45% respectively (3). The overall fracture incidence is as high as 10 to 25 per 1,000 patient-years (4). There is even report stating that 1 in 10 women aged 65 years and older will experience a fracture within 3 years of starting dialysis (5). The US Renal Data System (USRDS) has shown a four-fold increase in the risk of hip fractures among end-stage renal disease (ESRD) patients, and the mortality rate for hemodialysis patients after hip fracture is 2.7 times higher compared to those without hip fractures (6). One-year mortality rates can reach 64%, making it one of the leading causes of death among MHD patients (7). However, there is limited research on treatment choices for hip fractures in MHD patients, and the existing studies have small sample sizes and only provide simple comparisons of mortality rates, resulting in low credibility of conclusions.

Currently, it is widely believed that post-operative mortality and complication rates are significantly higher in MHD patients compared to non-hemodialysis patients (1). However, compared to conservative treatment, whether surgical treatment can improve the survival outcome of MHD patients with hip fracture remains to be verified. Other than that, whether surgical treatment can accelerate the recovery of joint function and shorten the time of bed rest after hip osteoporotic fracture in MHD patients? More importantly, is surgical treatment safe in the MHD population and does it increase the occurrence of complications after hip fracture is also worth exploring. Once MHD patients suffer from secondary hip osteoporotic fracture, it will significantly increase the risk of disability and death and lead to a huge global public health burden. Therefore, this study adopted a retrospective analysis method to investigate the efficacy and safety of surgical treatment in 43 patients with osteoporotic hip fractures undergoing MHD treatment, aiming to provide evidence-based proofs for clinical practice and offer potential opportunities for improving patient prognosis.

## 2 Materials and methods

### 2.1 Study design and participants

Retrospective analysis was conducted using clinical and ancillary examination data of MHD patients with hip fractures admitted to Tianjin Hospital of Tianjin University from August 2019 to August 2023. Inclusion criteria: ① Age ≥ 60 years, ② Hip osteoporotic fracture, including femoral neck fracture, intertrochanteric fracture, and subtrochanteric fracture, based on the results of dual-energy x-ray absorptiometry, osteoporosis was diagnosed in patients with a T-score of the hip ≤ 2.5, ③ Regular hemodialysis treatment for ≥ one month. Exclusion criteria: ① Hip fractures not associated with osteoporosis, ② Open fractures, ③ Multiple fractures or severe associated injuries resulting from severe traumas like car accidents or falls from buildings, ④ Concurrent hematological disorders, severe rheumatic diseases, or malignant tumors, ⑤ Lost to follow-up. This study was approved by the Ethics Committee of Tianjin Hospital of Tianjin University (Date: 2021.11.15, No. 2021YiLunShen155). All enrolled patients provided written informed consent and no details that might infringes the privacy rights of the subjects were disclosed.

### 2.2 Treatment methods

A total of 43 MHD patients with hip osteoporotic fractures were included, including 28 cases of femoral neck fracture, 11 cases of intertrochanteric fracture, and four cases of subtrochanteric fracture. There were 13 cases in the conservative group and 30 cases in the surgical treatment group. Conservative treatment mainly included immobilization and supracondylar traction of femur. The type of surgery (total hip arthroplasty, hemiarthroplasty, open reduction internal fixation, closed reduction internal fixation) was determined based on the patient's age, location and type of fracture, functional capacity, medical condition, and preference. In general, for intertrochanteric fracture and subtrochanteric fracture, closed reduction was preferred. If the reduction was satisfactory as observed by intraoperative fluoroscopy, screw fixation would be applied. Otherwise, open reduction internal fixation was performed. For patients with femoral neck fracture, according to the scores of quantitative score system for the surgical decision on adult femoral neck fracture (8), internal fixation was used for score of 1–11. For patients with score of 12–17 who are in good physical condition and have strong exercise capacity, total hip arthroplasty was adopted. For patients with score of 18–22 who have poor physiological conditions and poor exercise capacity, hemiarthroplasty was selected. A total of 16 cases of total hip arthroplasty, six cases of hemiarthroplasty, four cases of open reduction internal fixation, and four cases of closed reduction internal fixation were performed.

### 2.3 Observational indicators and follow-up focus

The following data were collected for the enrolled participants: age, gender, duration of dialysis, history of renal dysfunction, comorbidities (mainly including hypertension, diabetes, cardiovascular disease, chronic lung disease, cerebrovascular disease, neoplasms) and pre-admission or preoperative laboratory tests (hemoglobin, albumin, brain natriuretic peptide, parathormone, calcium, phosphate, beta-crosslaps, propeptide of type I procollagen, bone-specific alkaline phosphatase). We used the Charlson Comorbidity Index, which included 16 common comorbidities and was assigned according to the severity, to assess patients' comorbidities and baseline mortality risk. One month after the fracture, the Harris score is performed to assess the early joint function recovery in surviving patients. The score comprehensively evaluates multiple aspects including pain, daily activities, gait, the need for assistive devices, walking distance, and joint deformity. Additionally, the blood routine and liver function tests are conducted during the one-month follow-up after the fracture, with the assessment of nutritional status using total protein, hemoglobin, and the calculation of the prognostic nutritional index [PNI = serum albumin (g/L) + 5 × peripheral blood lymphocyte count (×10^9^/L)] to exclude interference. At three months after the fracture, the Barthel index is used to evaluate the late joint function recovery by assessing the patients' activities of independent daily living. All patients were followed up until 31 August 2023, and the following information was recorded: admission time, surgery time, follow-up time, time to first ambulation assisted by walking aid after fracture, occurrence, and timing of complications during the follow-up period, survival outcome, and time of death. The original follow-up data are included in the Supplementary Table S1.

### 2.4 Data analyses

Data analysis was performed using SPSS 25.0 statistical software. Continuous variables are presented as mean ± standard deviation or median (interquartile range). Group comparisons were performed using independent samples *t*-test or Mann-Whitney *U*-test. Categorical variables are presented as frequencies (percentages), and group comparisons were performed using *Chi*-square test. Patient survival rates were analyzed using Kaplan-Meier survival curves and the log-rank test. Multivariable Cox regression analysis was conducted to identify the risk factors influencing survival outcomes and the occurrence of complications in MHD patients with hip osteoporotic fractures. *P* < 0.05 indicates statistically significant differences.

## 3 Results

### 3.1 Baseline data of enrolled patients

Before comparing the effects of different treatment methods on survival outcomes, we first conducted statistical analysis on the demographic information, medical history, comorbidities and initial biochemical indicators of the patients included in each group, to confirm the comparability of data between the two groups. A total of 43 MHD patients with hip osteoporotic fractures were included. Of these, 19 men and 24 women, 13 patients were in the conservative treatment group, and 30 patients were in the surgical treatment group. No significant differences were observed between the two groups in terms of age, gender distribution, duration of dialysis, history of renal insufficiency, comorbidities, mortality risk, follow-up duration, and baseline levels of hemoglobin (Hb), albumin (ALB), brain natriuretic peptide (BNP), parathormone (PTH), calcium (Ca), phosphate (P), beta-crosslaps (*β*-CTX), propeptide of type I procollagen (PINP), and bone-specific alkaline phosphatase (BAP), as shown in Table 1.

**Table 1:** Comparison of baseline characteristics of enrolled patients.

Projects	Conservative treatment	Surgical treatment	*P*-value
Number of cases	13	30	–
Age (years)	72.92 ± 7.39	70.70 ± 8.06	0.400
Gender (Male/Female)	7/6	12/18	0.401
Duration of dialysis (month)	1 (53)	7 (35)	0.944
History of renal insufficiency (year)	6 (8.5)	5 (7.63)	0.801
Hypertension (number)	13	28	0.340
Diabetes (number)	6	16	0.665
Cardiovascular disease (number)	7	18	0.707
Chronic lung disease (number)	1	2	0.903
Cerebrovascular disease (number)	4	10	0.869
Neoplasms (number)	1	0	0.124
Charlson Comorbidity Index	7.46 ± 1.76	7.00 (2.25)	0.635
Follow-up duration	1.7 (23)	18 (28.75)	0.062
Hb	95.23 ± 16.49	103.27 ± 15.48	0.133
ALB	33.97 ± 5.56	31.36 ± 4.43	0.109
BNP	863.03 (1,903)	231.45 (307.70)	0.064
PTH	213.4 (289.35)	322.6 (341.75)	0.093
Ca	2.21 (0.21)	2.18 (0.3)	0.475
P	1.65 ± 0.38	1.79 ± 0.60	0.440
*β*-CTX	1.96 ± 0.82	2.32 ± 1.06	0.290
PINP	156.90 (67.50)	181.80 (69.40)	0.278
β-CTX/PINP	0.010 (0.004)	0.012 (0.008)	0.412
BAP	14.11 (7.02	14.44 (10.16)	0.902

### 3.2 Comparison of the differences in survival outcomes after Hip fractures in MHD patients treated with surgical vs. conservative treatments

**Figure 1:**
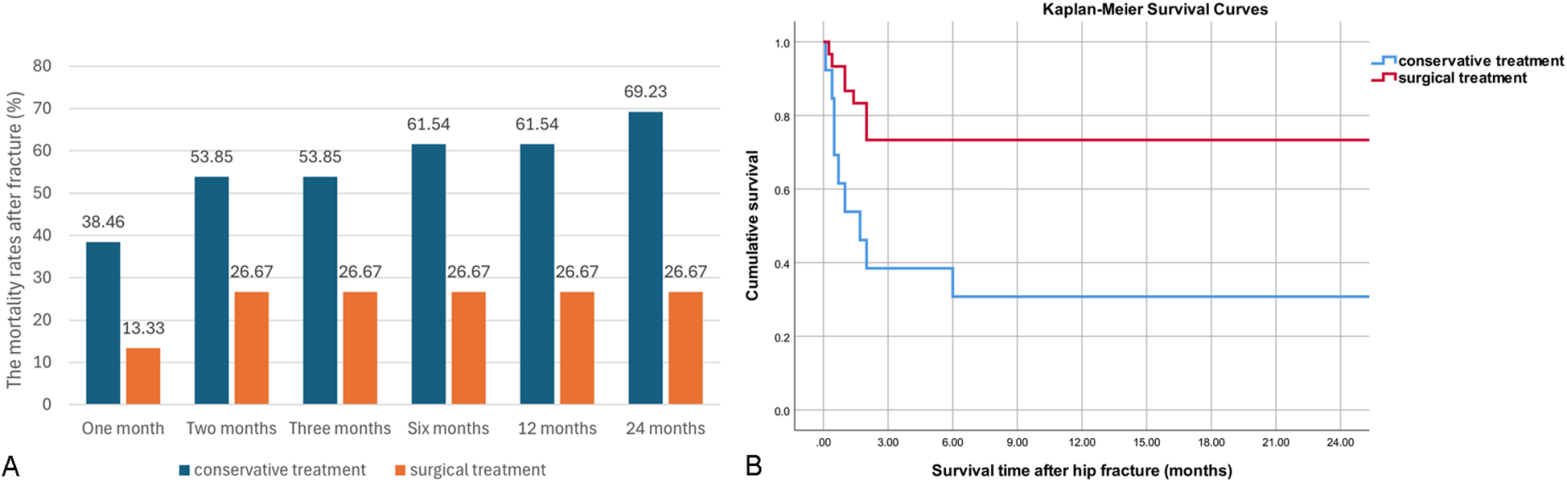
Comparison of the differences in survival outcomes between different treatment groups. **(A)** The mortality rates after fracture. The mortality rates after fracture between the different treatment groups are shown in the bar chart. The surgical group had remarkable lower mortality rates as compared with the conservative group at 1, 2, 3, 6, 12, 24 months (13.33 VS. 38.46%, 26.67 VS. 53.85%, 26.67 VS. 53.85%, 26.67 VS. 61.54%, 26.67 VS. 61.54%, and 26.67 VS. 69.23%). **(B)** Kaplan-Meier Survival Curves Analysis of conservative treatment and surgical treatment in MHD patients with hip osteoporotic fracture. The Kaplan-Meier method was used to estimate overall survival, and we conclude that: compared with conservative treatment, surgical treatment can significantly lower mortality rates, thereby prolonging survival.

To address the above question, we conducted continuous follow-up of patients and compared the mortality rates at different time points and post-fracture survival time. The surgical group had remarkable lower mortality rates as compared with the conservative group at all time-points, suggesting that proper surgical treatment in the MHD population is safe and feasible. The median survival time for patients in the conservative group was 1.7 months, while it was 18 months for patients in the surgical treatment group. The mortality rates at 1, 2, 3, 6, 12, and 24 months after fracture in both groups are shown in Figure 1A. The Log-rank test revealed a statistically significant difference in survival rates between the two groups (*P* = 0.004), as depicted in Figure 1B using the Kaplan-Meier survival curve. Multivariable Cox regression analysis of survival outcome is shown in Table 2. The results revealed that the risk of death was significantly reduced to only 23.0% of conservative treatment by proper surgical treatment in MHD patients with hip fracture (*P* = 0.004). In addition to treatment modality, a comprehensive analysis of age, gender, duration of dialysis, history of renal insufficiency, comorbidities, and baseline levels of Hb, ALB, BNP, PTH, Ca, P, *β*-CTX, PINP, and BAP showed that only increasing age significantly increased the risk of mortality. For each year increase in age, the risk of mortality after hip osteoporotic fracture in MHD patients increased by 1.122 times (95% confidence interval: 1.052-1.197).

**Table 2:** Multivariable cox regression analysis of survival outcome.

Projects	*P*-value	Regression coefficients	Relative risk (95% confidence interval)
Whether received operation	0.004	−1.468	0.230（0.085∼0.625）
Age	<0.001	0.115	1.122（1.052∼1.197）

### 3.3 Differences in joint function recovery after hip fractures among MHD patients with different treatments

**Figure 2:**
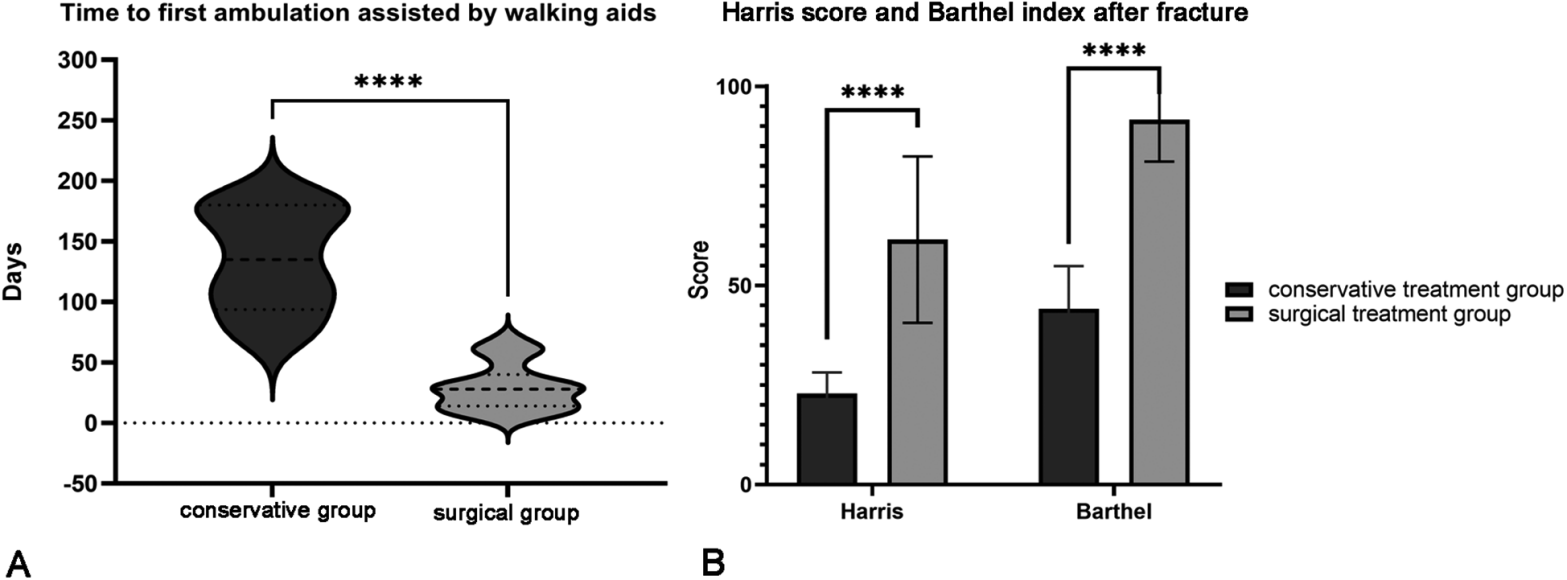
Hip joint functional recovery between different treatment groups. **(A)** Comparison of the time to first ambulation after fracture. Mann-Whitney *U*-test indicated that surgical treatment effectively shortened the first ambulation to 28 (26) days, which was superior to the conservative group (134.17 ± 43.18 days). **(B)** Comparison of Harris score and Barthel index after fracture. The Harris score at one month after the fracture and the Barthel index at three months after the fracture were performed to reflect early joint function recovery and long-term daily activity capability, respectively. The above scores in the surgical treatment group were significantly higher than those in the conservative treatment group, indicating that appropriate surgical treatment did accelerate the recovery of joint function in MHD parents.

In the conservative treatment group, the time to first ambulation assisted by walking aids after fracture showed a normal distribution, with an average of 134.17 ± 43.18 days. In the surgical treatment group, the time to first ambulation assisted by walking aids after fracture did not follow a normal distribution, with a median time of 28 days. Mann-Whitney *U*-test indicated that surgical treatment effectively shortened the first ambulation, which was superior to the conservative group (*P* < 0.001, Figure 2A). Subsequently, in this study, the Harris score at one month after the fracture and the Barthel index at three months after the fracture were performed to reflect early joint function recovery and long-term daily activity capability, respectively. The Harris score (61.50 ± 4.10) and Barthel index (95.11 ± 11.25) of the surgical treatment group were significantly higher than those of the conservative treatment group (*P* < 0.001, Figure 2B), indicating that appropriate surgical treatment indeed accelerated the recovery of joint function. To eliminate the impact of differences in nutritional status on joint function recovery, we compared the PNI, total protein, and hemoglobin levels of the two groups of patients at one month after the fracture. The differences were not statistically significant (Table 3).

**Table 3:** The nutritional status at one month after the fracture.

	Conservative treatment	Surgical treatment
PNI (*P* = 0.583)	39.35, 4.65	38.35 ± 0.88
Total protein (*P* = 0.990)	60.47 ± 4.16	60.43 ± 0.91
Hemoglobin (*P* = 0.580)	95.00 ± 4.74	98.11 ± 2.76

### 3.4 Comparison of the incidence of complications after hip fractures in MHD patients treated with surgical vs. conservative treatments

**Figure 3:**
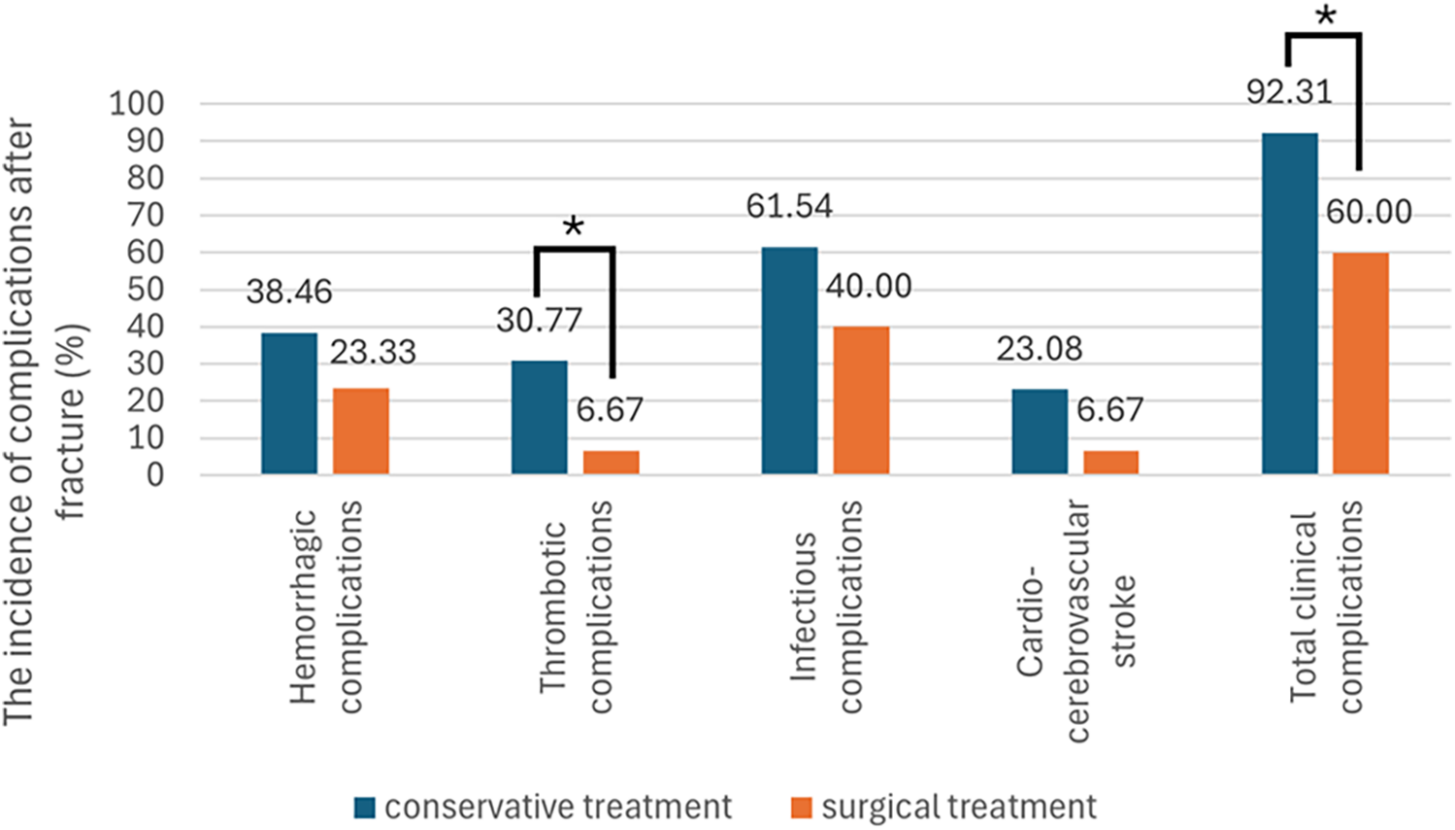
Comparison of the incidence of complications after hip fractures between different treatment groups. The incidence of complications after fracture in patients of different treatment groups is shown in the bar chart. According to statistics, the incidence of thrombotic complications (6.67%, *P* = 0.036) and overall clinical complications (60.00%, *P* = 0.034) in the follow-up period were significantly lower in the surgical treatment group with statistically significant differences.

The occurrence of complications in different treatment groups is shown in Table 4, the incidence of clinical complications after fracture was presented as a bar chart (Figure 3). The results of the analysis showed that the incidence of thrombotic complications and overall clinical complications in the follow-up period were significantly lower in the surgical treatment group with statistically significant differences. Among the 30 MHD patients who underwent surgical treatment, a total of four cases had surgical-related complications, with a cumulative incidence rate of 13.33%. These complications included one case of joint dislocation, one case of periprosthetic fracture, and two cases of local hematoma at the surgical site. The multivariate Cox regression analysis (Table 5) showed that surgical treatment significantly reduced the risk of post-fracture clinical complications to 32.4% compared with the conservative treatment (*P* = 0.005). Besides treatment modality, the comprehensive analysis of patient age, gender, duration of dialysis, history of renal insufficiency, comorbidities, baseline routine, biochemical, and bone turnover markers revealed that only increasing age significantly increased the risk of post-fracture complications. For each year increase in age, the risk of clinical complications (including secondary infections, bleeding, thrombosis, cardiovascular and cerebrovascular events) increased by 1.057 times (95% confidence interval: 1.010-1.106) after hip osteoporotic fractures in MHD patients. All the above suggest that proper surgical treatment of osteoporotic hip fractures in MHD patients is safe and effective, which is superior to conservative treatment.

**Table 4:** The occurrence of complications.

Complications	Conservative treatment		Surgical treatment	
	Cases/constituent ratio (%)	Incidence (%)	Cases/constituent ratio (%)	Incidence (%)
Pulmonary infectionss	7（58.33）	53.85	10（41.66）	33.33
Lower limb venous thrombosis	4（33.33）	30.77	2（8.33）	6.67
Cerebral hemorrhage	3（25）	23.08	/	/
Gastrointestinal bleeding	2（16.67）	15.38	6（25）	20
Myocardial infarction	2（16.67）	15.38	/	/
Acute cerebral infarction	1（8.33）	7.69	2（8.33）	6.67
Urinary tract infection	1（8.33）	7.69	2（8.33）	6.67
Subcutaneous bleeding	/	/	1（4.17）	3.33
Metabolic encephalopathy	/	/	1（4.17）	3.33

**Table 5:** Multivariate cox regression analysis of complications.

Projects	*P* value	Regression coefficients	Relative risk (95%confidence interval)
Whether received operation	0.005	−1.126	0.324（0.147∼0.717）
Age	0.017	0.055	1.057（1.010∼1.106）

## 4 Discussion

According to the data from the USRDS, hip fractures significantly increase the risk of mortality in MHD patients, with a one-year mortality rate of up to 64% (6, 7). It has gradually become one of the leading causes of death among MHD patients. In terms of treatment, previous literature has reported that delaying surgery in hip fracture patients increases the 30-day mortality rate by 2.78 times in the general population (9). Early surgery is beneficial in reducing hospitalization time, lowering medical costs, significantly improving joint function, and decreasing mortality rates. However, for the population on MHD, it is generally believed that there is a significantly higher risk of readmission, revision surgery, and mortality compared to non-dialysis-dependent individuals (10–12). Furthermore, based on the important role of the kidneys in maintaining bone mineralization and bone turnover, the occurrence rate of osteoporosis is higher in MHD patients. Osteoporosis also decreases the stability of fracture fixation, thus affecting the outcomes of surgical treatment (12). Therefore, the choice between conservative non- surgical treatment and surgical treatment for hip osteoporotic fractures in MHD patients remains a topic of debate. The article starts by using mortality at different time points and cumulative survival time after fracture as core observational indicators, confirming that even with increased surgical risks in MHD patients, reasonable surgical treatment can still significantly improve the survival outcomes after hip fracture compared to conservative treatment. Previous studies have found that risk factors for mortality after hip fractures in the elderly population mainly include advanced age, male gender, residing in nursing homes, poor preoperative ambulatory capacity, poor daily activity capacity, poor mental status, and comorbidities (13). In this study, we also found that advanced age is another risk factor for mortality, independent of the treatment method, which is consistent with previous research. Previous studies, after adjusting for traditional risk factors of hemodialysis, have found that hypoparathyroidism is an independent predictor of overall mortality and cardiovascular mortality in MHD patients with hip fractures (14). Low PTH levels may be accompanied by aggravated vascular calcification, leading to cardiovascular death. However, in our study, due to the small baseline differences in PTH levels among the enrolled patients in this study, no significant differences were observed when comparing the PTH levels between patients who died after the fracture and those who survived. Therefore, the impact of PTH on the risk of mortality in MHD patients with osteoporotic hip fractures could not be observed. The study also found that regardless of conservative or surgical treatment, the majority of deaths in MHD patients with osteoporotic hip fractures occurred within three months after the fracture, which is consistent with the temporal pattern of post-fracture complications.

In addition to survival outcomes, for patients with fractures, the recovery of joint function and the subsequent capacity for action are also the focus of our attention. This study proposes that surgical treatment shortens the bedridden time in MHD patients with osteoporotic hip fractures. Excluding the interference of nutritional status, earlier ambulation time, higher Harris score and Barthel index after fracture support that surgical treatment is superior to conservative treatment in the recovery of joint function. The shortening of bedridden time, the fast recovery of hip joint function and the improvement in activities of daily living in long-term follow-up all suggest that, compared with conservative treatment, surgical treatment significantly improves the quality of life of patients. Surgical treatment is more conducive to patients' return to society while reducing the economic and psychological burdens of patients. In addition, we also found that there are differences in the incidence and composition of complications in MHD patients with hip fractures treated with different treatment methods. The top five complications in the conservative treatment group, in descending order, were pulmonary infection, lower limb deep vein thrombosis, cerebral hemorrhage, gastrointestinal bleeding, and myocardial infarction. In the surgical treatment group, the top five complications were pulmonary infection, gastrointestinal bleeding, lower limb deep vein thrombosis, cerebral infarction, and urinary tract infection. Conservative treatment and advanced age were significant risk factors for the occurrence of complications following hip fractures in MHD patients with osteoporosis. The incidence of thrombotic complications and total clinical complications during the follow-up period were significantly lower in the surgical treatment group. Furthermore, the proportion of lower limb deep vein thrombosis and bleeding events in the surgical treatment group was significantly lower than that in the conservative group. The reduction in thrombotic events is believed to be related to the earlier ambulation with the assistance of walking aids, which significantly reduces the risk of lower limb venous thrombosis and lowers the dosage of anticoagulants, thus reducing the occurrence of hemorrhagic events (15). For patients who undergo surgical treatment, although MHD patients may have a decreased immune response, the incidence of deep tissue infection in the surgical site did not occur in our patients, which is consistent with previous reports (16). For postoperative patients, readmission or reoperation is associated with higher mortality rates, with a one-year mortality rate of 35 to 48% (17–22). Lan et al. 's meta-analysis mentioned that compared to non-dialysis-dependent patients, MHD patients have a significantly higher reoperation rate for revision surgery after fractures (1). This may be associated with more postoperative complications and a state of poor bone nutrition. In our study, among 30 patients with maintenance hemodialysis and hip fractures who underwent surgical treatment, a total of four patients experienced surgery-related complications. These included one case of joint dislocation, one case of periprosthetic fracture, and two cases of localized hematoma at the surgical site. Among these cases, only one patient underwent reoperation and died within one month after the reoperation. The lower incidence of surgery-related complications in this study may be attributed to the fact that a majority of the patients included in the surgical group underwent total hip arthroplasty/hemiarthroplasty following femoral neck fractures. Previous research has found that patients with femoral neck fractures may have better survival outcomes compared to patients with intertrochanteric fractures in the general population (12). This may be due to earlier ambulation with the assistance of walking aids and reduced bed rest-related complications in patients who underwent total joint arthroplasty compared to internal fixation procedures. In the dialysis population, studies have also compared internal fixation, screw fixation, and hemiarthroplasty, and found a significant reduction in the occurrence of complications following hemiarthroplasty (23). MHD patients often experience disturbances in bone metabolism and various comorbidities, which increase the risk of nonunion and avascular necrosis following internal fixation procedures, leading to a higher rate of readmission for revision surgery (24). Patients on maintenance hemodialysis have a significantly high occurrence rate of *β*2-microglobulin amyloidosis, which leads to the deposition of amyloid substances that destroy normal bone and cause osteolytic bone destruction. The femoral neck, scaphoid bone, and C1-C2 vertebrae are the most commonly affected sites (25). There are also studies that mention the occurrence of secondary hyperparathyroidism and intra- and extra-articular *β*2-microglobulin amyloidosis in MHD patients significantly increases the risk of prosthetic loosening following hemiarthroplasty (26). We will continue to monitor and further investigate the differences in treatment outcomes among different fracture types and surgical procedures.

As a retrospective study, there may be some selection biases such as survivorship bias and bias caused by economic factors. However, for this study, first of all, we did not ignore patients who died and those who withdrew due to other factors. On the contrary, the occurrence of death itself is an important observation endpoint of this study. We used Kaplan-Meier survival curves and the log-rank test to simultaneously consider the survival time and occurrence of death, and multivariate Cox regression analysis was used to further confirm the significant impact of treatment selection on survival outcomes. In terms of economic factors, neither conservative treatment nor surgical treatment involves self-pay items. And with the improvement of China's medical security system, the cost of surgical treatment has been decreasing year by year in recent years, which doesn't cause a significant economic burden. At the same time, the expected length of hospital stay for patients with conservative treatment is significantly prolonged, and the additional hospitalization costs and nursing costs will also narrow the cost difference between the two treatment methods. In addition, there is no significant difference in baseline data including age, duration of dialysis, comorbidities and biochemical indicators between the two groups of patients. All of the above support that the patients' data is comparable and the research results are true and can be promoted.

There are several limitations in this study: Although we statistically analyzed the baseline Ca, P, PTH, *β*-CTX, PINP, and BAP, confirming the comparability among groups, we did not continuously track these indicators during the follow-up period. Other than that, clinical observational studies and animal experiments have also mentioned that certain medications, such as proton pump inhibitors, antidepressants, anticoagulants, and high levels of erythropoietin use, may increase the risk of fractures in maintenance hemodialysis patients (27). However, whether the use of these drugs after fractures would affect fracture healing and interfere with the evaluation of surgical outcomes still requires further exploration. Our study also lacks imaging results for the evaluation of hip joint recovery, especially the extent of periprosthetic bone resorption. In the future, we will establish ongoing collaboration with the surgical department to supplement data on intraoperative conditions and postoperative recovery, continuously increase the sample size, and enhance the strength of evidence for our conclusions.

Based on the important role of the kidneys in maintaining mineral-bone stability and the process of bone remodeling, the maintenance hemodialysis state is significantly associated with an increased risk of hip osteoporotic fractures. Moreover, the mortality rate after fractures is extremely high, making it one of the main causes of death in MHD patients. Proper surgical treatment of osteoporotic hip fractures in maintenance hemodialysis population is safe and effective. Compared with conservative treatment, surgical treatment can significantly reduce the mortality rate and prolong survival after hip osteoporotic fractures in MHD patients. It also effectively shortens bed rest time, accelerates joint function recovery and reduces the occurrence of complications. In summary, for patients with maintenance hemodialysis and concomitant hip fracture, after excluding surgical contraindications, surgical treatment is the first choice for clinical physicians.

## Data Availability

The original contributions presented in the study are included in the article/Supplementary Material, further inquiries can be directed to the corresponding author.
